# Supplementary material for: Influence of Molecular Structure and Physicochemical Properties of Immunosuppressive Drugs on Micelle Formulation Characteristics and Cutaneous Delivery
Source: Pharmaceutics. 2023 Apr 19;15(4):1278. doi: 10.3390/pharmaceutics15041278 (PMC10142028; doi:10.3390/pharmaceutics15041278)
Supplement: Supplementary file 1 [file pharmaceutics-15-01278-s001.zip › pharmaceutics-2259328-supplementary.pdf]

Supplementary Materials: Influence of molecular structure and physicochemical properties of immunosuppressive drugs on micelle formulation characteristics and cutaneous delivery

Julie Quartier, Maria Lapteva, Younes Boulaguiem, Stéphane Guerrier and Yogeshvar N. Kalia *

Analytical method for PIM

Validation of UHPLC-UV method

Specificity

The method was considered specific for PIM. Indeed, PIM was eluted at 0.80 min and the peaks due to the polymer appeared at 2.78 min (for TPGS) and at 4.21 min (for α-tocopherol). The chromatograms in Figure S1 present the signals of PIM standard (40 µg/mL), ACN blank, PIM loaded in micelles and unloaded micelles.

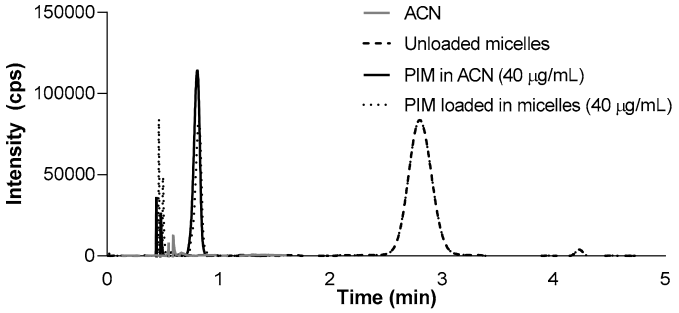

Figure S1. Chromatograms of ACN blank, unloaded micelles, PIM standard (40 µg/mL) and PIM loaded in micelles.

Linearity

Calibration curves were constructed by plotting PIM peak area (cps/min) against PIM nominal concentration (ng/mL). A good linear fit was found in the concentration range of 5 – 150 µg/mL. Correlation coefficients for all calibration curves were superior to 0.99.

Limit of detection and limit of quantification

The limit of detection (LOD) and the limit of quantification (LOQ) of PIM were found to be 1.60 and 5.00 µg/mL, respectively.

Precision and accuracy

Intra- and inter-day precision and accuracy were tested using 5, 20, 80 µg/mL of PIM in ACN. Table S1 presents the results obtained for intra- and inter-day precision and accuracy.

Table S1. Intra- and inter-day precision and accuracy for quantification of PIM in ACN with UHPLC-UV.

	Intra-day			Inter-day 1			Inter-day 2		
$[PIM]_{theo}$ (µg/mL)	$[PIM]_{meas}$ (µg/mL)	RSD (%)	Recovery (%)	$[PIM]_{meas}$ (µg/mL)	RSD (%)	Recovery (%)	$[PIM]_{meas}$ (µg/mL)	RSD (%)	Recovery (%)
5	4.36 ± 0.01	0.19	87.4	4.55 ± 0.27	5.87	91.2	5.00 ± 0.27	5.49	99.9
20	18.52 ± 1.15	6.18	92.6	19.41 ± 1.18	6.06	97.0	20.89 ± 0.69	3.30	104.5
80	76.86 ± 3.06	3.98	96.1	78.77 ± 3.35	4.26	98.5	77.40 ± 3.16	4.08	96.8

The results indicated that for intra-day measurements, the mean recoveries ranged from 87.4 to 96.1 (RSD 0.19 – 6.18%). The mean recoveries for inter-day analysis on day 1 were between 91.2 and 98.5 (RSD 4.26 – 6.06%) and on day 2, 96.8 and 104.5 (RSD 3.30 – 5.49%). Considering the ICH (2005) and FDA Bioanalytical Method Validation (2001) guidelines, the method was considered accurate and precise.

Validation of UHPLC-MS/MS method in ACN

Specificity

The chromatograms in Figure S2 present the MS signal of PIM and TAC standard alone and mixture of the compounds prepared in ACN.

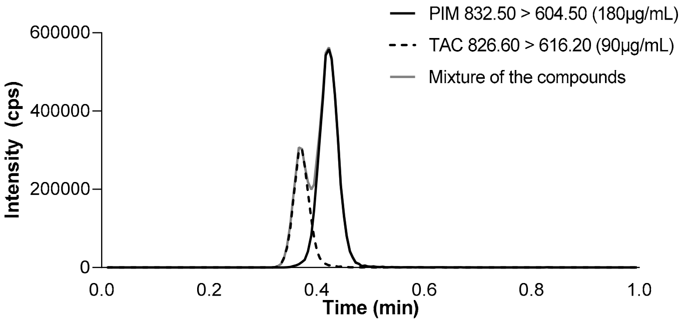

Figure S2. Respective MRM traces on XEVO TQ MS of **a)** PIM 832.50 > 604.50, **b)** TAC 826.60 > 616.20 and **c)** mixture of compounds prepared in ACN.

Linearity

Calibration curves were constructed by plotting the ratio of PIM and TAC peak area (cps/min) against the ratio of PIM and TAC nominal concentration (ng/mL). A good linear fit was found in the concentration range of 0.03 – 2.5 (PIM/TAC concentration ratio), that corresponds from 225 to 3 ng/mL of PIM. Correlation coefficients for all calibration curves were superior to 0.99.

Limit of detection and limit of quantification

The limit of detection (LOD) and the limit of quantification (LOQ) of PIM were found to be 1 and 3 ng/mL, respectively.

Precision and accuracy

Intra- and inter-day precision and accuracy were tested using 3, 12, 120 ng/mL of PIM in ACN. Table S2 presents the results obtained for intra- and inter-day precision and accuracy.

Table S2. Intra- and inter-day precision and accuracy for quantification of PIM in ACN with XEVO TQ MS.

	Intra-day			Inter-day 1			Inter-day 2		
$[PIM]_{theo}$ (ng/mL)	$[PIM]_{meas}$ (ng/mL)	RSD (%)	Recovery (%)	$[PIM]_{meas}$ (ng/mL)	RSD (%)	Recovery (%)	$[PIM]_{meas}$ (ng/mL)	RSD (%)	Recovery (%)
3	3.13 ± 0.11	3.41	104.4	2.71 ± 0.04	1.31	90.2	3.21 ± 0.21	6.61	107.0
12	11.83 ± 0.39	3.32	98.6	11.16 ± 0.31	2.75	93.0	12.42 ± 0.57	4.56	103.5
120	122.08 ± 1.95	1.60	101.7	117.55 ± 3.04	3.58	98.0	124.43 ± 5.25	4.22	103.7

The results indicated that for intra-day measurements, the mean recoveries ranged from 98.6 to 104.4 (RSD 1.60 – 3.41%). The mean recoveries for inter-day analysis on day 1 were between 90.2 and 98.0 (RSD 1.31 – 3.58%) and on day 2,103.5 and 107.0 (RSD 4.22 – 6.61%). Considering the ICH (2005) and FDA Bioanalytical Method Validation (2001) guidelines, the method was considered accurate and precise.

Validation of UHPLC-MS/MS method in skin matrix

Specificity

The chromatograms in Figure S3 present the MS signal of PIM, TAC and mixture of the compounds prepared in skin matrix.

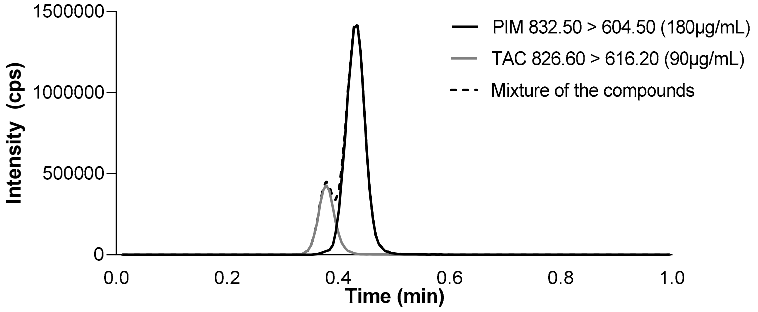

Figure S3. Respective MRM traces on XEVO TQ MS of **a)** PIM 832.50 > 604.50, **b)** TAC 826.60 > 616.20 and **c)** mixture of compounds prepared in skin matrix.

Figure S4 presents the chromatograms obtained for PIM standard, blank permeation and blank extraction sample. No signal at the PIM elution time was found in blank skin permeation and extraction samples.

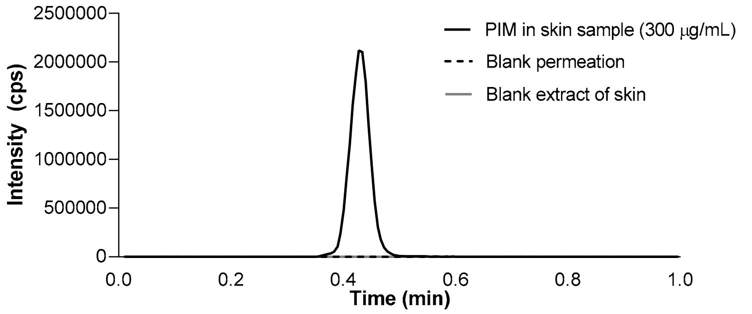

Figure S4. Respective MRM traces of PIM in skin samples (832.50 > 604.50): **a)** PIM in matrix, **b)** blank permeation and **c)** blank extract of skin.

The method was considered as specific for PIM quantification in skin samples.

Linearity

Calibration curves were constructed by plotting the ratio of PIM and TAC peak area (cps/min) against the ratio of PIM and TAC nominal concentration (ng/mL). A good linear fit was found in the concentration range of 0.03 – 2.5 (PIM/TAC concentration ratio), that corresponds from 225 to 3 ng/mL of PIM. Correlation coefficients for all calibration curves were superior to 0.99.

Limit of detection and limit of quantification

The limit of detection (LOD) and the limit of quantification (LOQ) of PIM were found to be 1 and 3 ng/mL, respectively.

Precision and accuracy

Intra- and inter-day precision and accuracy was tested using 3, 12, 120 ng/mL of PIM in skin matrix. Table S3 presents the results obtained for intra- and inter-day precision and accuracy.

Table S3. Intra- and inter-day precision and accuracy for quantification of PIM in skin matrix with XEVO TQ MS.

	Intra-day			Inter-day 1			Inter-day 2		
$[PIM]_{theo}$ (ng/mL)	$[PIM]_{meas}$ (ng/mL)	RSD (%)	Recovery (%)	$[PIM]_{meas}$ (ng/mL)	RSD (%)	Recovery (%)	$[PIM]_{meas}$ (ng/mL)	RSD (%)	Recovery (%)
3	3.05 ± 0.21	7.16	101.6	2.89 ± 0.29	9.89	96.4	3.05 ± 0.39	12.67	101.7
12	11.38 ± 0.69	6.05	94.8	12.04 ± 0.72	5.98	100.3	11.42 ± 1.06	9.31	95.2
120	125.12 ± 3.17	2.62	100.9	125.01 ± 3.67	2.93	104.2	118.17 ± 7.68	6.50	98.5

The results indicated that for intra-day measurements, the mean recoveries ranged from 94.8 to 101.6 (RSD 2.62 – 7.16%). The mean recoveries for inter-day analysis on day 1 were between 96.4 and 104.2 (RSD 5.98 and 9.89%) and on day 2, 95.2 and 101.7 (RSD 6.50 – 12.67%). Considering the ICH (2005) and FDA Bioanalytical Method Validation (2001) guidelines, the method was considered accurate and precise.

Validation of skin extraction procedure

The ability of the extraction methods to recover the entire PIM deposited amount during *in vitro* permeation experiments was tested.

Validation of PIM extraction from total skin samples

Each skin sample (n = 3; 2 cm^2) was spiked with a certain concentration of PIM (in acetone). After acetone evaporation, skin samples were cut into small pieces and soaked in 4 mL of ACN with internal standard (TAC) and left overnight. The skin extracts were then analyzed by UHPLC-MS/MS and calibration curve was prepared in ACN. The procedure extraction of total skin was validated as presented in Table S4

Table S4. Validation of PIM extraction from total skin samples.

Applied amount/cm^2 (μg)	Recovered amount/cm^2 (Mean ± SD in μg)	Recovery (%)
14	14.85 ± 0.67	106.08
4	4.49 ± 0.37	112.48
0.1	0.11 ± 0.01	109.01

For all skin samples 100% of PIM was recovered during the extraction procedure.

Validation of PIM extraction from sliced skin samples

Each skin sample was snap-frozen in isopentane cooled to its freezing point (-160°C) with liquid nitrogen and mounted in the cryotome. The samples were sliced so as to obtain 40 μm slices. Each slice (n = 3; 0.8 cm^2) was spiked with a certain concentration of PIM (in acetone). After acetone evaporation, slices were extracted in 300 μL of ACN with internal standard (TAC) and left overnight. The skin extracts were then analyzed by UHPLC-MS/MS and calibration curve was prepared in ACN. The procedure extraction of total skin was validated as presented in Table S5.

Table S5. Validation of PIM extraction from sliced skin samples.

Applied amount/cm^2 (ng)	Recovered amount/cm^2 (Mean ± SD in ng)	Recovery (%)
62.5	72.33 ± 4.06	115.74

12.5	14.23 ± 1.00	113.85
2.5	1.91 ± 0.62	91.27

For all skin samples more than 90% of PIM was recovered during the extraction procedure.

Analytical method for TAC

Validation of UHPLC-UV method

Specificity

The method was considered specific for TAC. Indeed, TAC was eluted at 0.66 min and the peaks due to the polymer appeared at 2.78 min (for TPGS) and at 4.21 min (for α-tocopherol). The chromatograms in Figure S5 present the signals of TAC standard (40 µg/mL), ACN blank, TAC loaded in micelles and unloaded micelles.

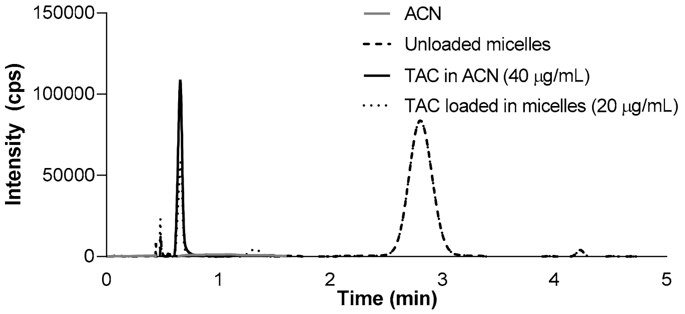

Figure S5. Chromatograms of ACN blank, unloaded micelles, TAC standard (40 µg/mL) and TAC loaded in micelles.

Linearity

Calibration curves were constructed by plotting TAC peak area (cps/min) against TAC nominal concentration (ng/mL). A good linear fit was found in the concentration range of 5 – 150 µg/mL. Correlation coefficients for all calibration curves were superior to 0.99.

Limit of detection and limit of quantification

The limit of detection (LOD) and the limit of quantification (LOQ) of TAC were found to be 0.7 and 2.0 µg/mL, respectively.

Precision and accuracy

Intra- and inter-day precision and accuracy were tested using 5, 20, 80 µg/mL of TAC in ACN. Table S6 presents the results obtained for intra- and inter-day precision and accuracy.

Table S6. Intra- and inter-day precision and accuracy for quantification of TAC in ACN with UHPLC-UV.

	Intra-day			**Inter-day 1**			**Inter-day 2**		
$[TAC]_{theo}$ (µg/mL)	$[TAC]_{meas}$ (µg/mL)	RSD (%)	Recovery (%)	$[TAC]_{meas}$ (µg/mL)	RSD (%)	Recovery (%)	$[TAC]_{meas}$ (µg/mL)	RSD (%)	Recovery (%)
2	1.81 ± 0.27	14.75	90.4	2.25 ± 0.15	6.63	112.6	2.75 ± 0.12	4.25	91.6
20	20.27 ± 0.35	1.74	101.4	19.06 ± 0.27	1.40	95.3	19.64 ± 0.13	0.64	98.2
80	80.49 ± 1.16	1.45	100.6	76.85 ± 0.98	1.28	96.1	77.57 ± 1.30	1.67	97.0

The results indicated that for intra-day measurements, the mean recoveries ranged from 90.4 to 101.4 (RSD 1.45 – 14.75%). The mean recoveries for inter-day analysis on day 1 were between 95.3 and 112.6 (RSD 1.28 – 6.63%) and on day 2, 91.6 and 97.0 (RSD 0.64 – 4.25%). Considering the ICH (2005) and FDA Bioanalytical Method Validation (2001) guidelines, the method was considered accurate and precise.

Validation of UHPLC-MS/MS method in ACN

Specificity

The chromatograms in Figure S6 present the MS signal of TAC and PIM internal standard alone and mixture of the compounds prepared in ACN.

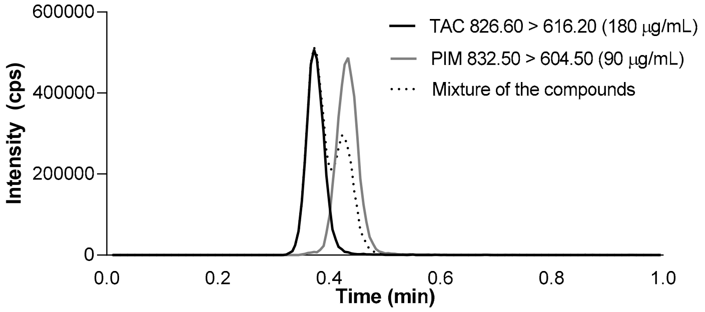

Figure S6. Respective MRM traces on XEVO TQ MS of **a)** TAC 826.60 > 616.20, **b)** PIM 832.50 > 604.50 and **c)** mixture of compounds prepared in ACN.

Linearity

Calibration curves were constructed by plotting the ratio of TAC and PIM peak area (cps/min) against the ratio of PIM and TAC nominal concentration (ng/mL). A good linear fit was found in the concentration range of 0.03 – 2.5 (TAC/PIM concentration ratio), that corresponds from 225 to 3 ng/mL of TAC. Correlation coefficients for all calibration curves were superior to 0.99.

Limit of detection and limit of quantification

The limit of detection (LOD) and the limit of quantification (LOQ) of TAC were found to be 1 and 3 ng/mL, respectively.

Precision and accuracy

Intra- and inter-day precision and accuracy were tested using 3, 12, 120 ng/mL of TAC in ACN. Table S7 presents the results obtained for intra- and inter-day precision and accuracy.

Table S7. Intra- and inter-day precision and accuracy for quantification of TAC in ACN with XEVO TQ MS.

	Intra-day			Inter-day 1			Inter-day 2		
$[TAC]_{theo}$ (ng/mL)	$[TAC]_{meas}$ (ng/mL)	RSD (%)	Recovery (%)	$[TAC]_{meas}$ (ng/mL)	RSD (%)	Recovery (%)	$[TAC]_{meas}$ (ng/mL)	RSD (%)	Recovery (%)
3	2.82 ± 0.20	7.31	94.2	2.74 ± 0.25	9.35	91.5	2.53 ± 0.18	7.20	84.2
12	12.39 ± 0.14	1.13	103.3	12.22 ± 0.50	4.12	101.8	11.51 ± 0.71	6.16	95.9
120	121.50 ± 2.18	1.80	101.2	122.68 ± 4.68	3.81	102.2	136.41 ± 6.61	4.85	113.7

The results indicated that for intra-day measurements, the mean recoveries ranged from 94.2 to 103.3 (RSD 1.13 – 7.31%). The mean recoveries for inter-day analysis on day 1 were between 91.5 and 102.2 (RSD

3.81 – 9.35%) and on day 2, 84.2 and 113.7 (RSD 4.85 – 7.20%). Considering the ICH (2005) and FDA Bioanalytical Method Validation (2001) guidelines, the method was considered accurate and precise.

Validation of UHPLC-MS/MS method in skin matrix

Specificity

The chromatograms in Figure S7 present the MS signal of TAC, PIM and mixture of the compounds prepared in skin matrix.

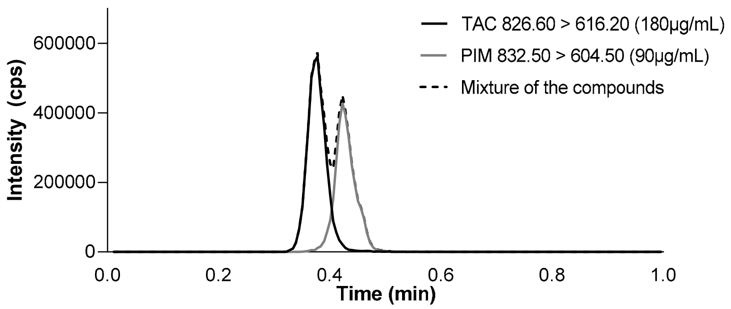

Figure S7. Respective MRM traces on XEVO TQ MS of **a)** TAC 826.60 > 616.20, **b)** PIM 832.50 > 604.50and **c)** mixture of compounds prepared in skin matrix.

Figure S8 presents the chromatograms obtained for TAC standard, blank permeation and blank extraction sample. No TAC was found in blank skin permeation and extraction samples.

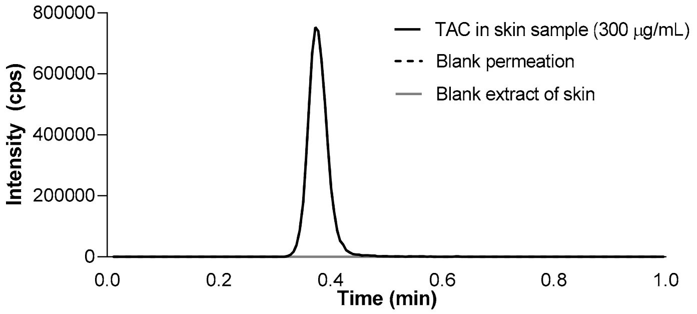

Figure S8. Respective MRM traces of TAC in skin samples (832.50 > 604.50): **a)** TAC in matrix, **b)** blank permeation and **c)** blank extract of skin.

The method was considered as specific for TAC quantification in skin samples.

Linearity

Calibration curves were constructed by plotting the ratio of TAC and PIM peak area (cps/min) against the ratio of TAC and PIM nominal concentration (ng/mL). A good linear fit was found in the concentration range of 0.03 – 2.5 (TAC/PIM concentration ratio), that corresponds from 225 to 3 ng/mL of TAC. Correlation coefficients for all calibration curves were superior to 0.99.

Limit of detection and limit of quantification

The limit of detection (LOD) and the limit of quantification (LOQ) of TAC were found to be 1 and 3 ng/mL, respectively.

Precision and accuracy

Intra- and inter-day precision and accuracy were tested using 3, 12, 120 ng/mL of TAC in skin matrix. Table S8 presents the results obtained for intra- and inter-day precision and accuracy.

Table S8. Intra- and inter-day precision and accuracy for quantification of TAC in skin matrix with XEVO TQ MS.

	Intra-day			Inter-day 1			Inter-day 2		
$[TAC]_{theo}$ (ng/mL)	$[TAC]_{meas}$ (ng/mL)	RSD (%)	Recovery (%)	$[TAC]_{meas}$ (ng/mL)	RSD (%)	Recovery (%)	$[TAC]_{meas}$ (ng/mL)	RSD (%)	Recovery (%)
3	3.08 ± 0.11	3.70	102.7	3.21 ± 0.14	4.28	107.0	2.71 ± 0.09	3.40	90.5
12	13.27 ± 0.53	3.98	110.6	13.10 ± 0.16	1.20	109.1	10.21 ± 0.23	3.65	85.1
120	130.06 ± 4.27	3.28	108.4	133.40 ± 5.00	2.93	104.2	109.91 ± 14.34	13.04	91.9

The results indicated that for intra-day measurements, the mean recoveries ranged from 102.7 to 110.6 (RSD 3.28 – 3.98%). The mean recoveries for inter-day analysis on day 1 were between 104.2 and 107.0 (RSD 1.20 – 4.28 %) and on day 2, 85.1 and 91.9 (RSD 3.40 – 13.04%). Considering the ICH (2005) and FDA Bioanalytical Method Validation (2001) guidelines, the method was considered accurate and precise.

Validation of skin extraction procedure

The ability of the extraction methods to recover the entire TAC deposited amount during *in vitro* permeation experiments was tested.

Validation of TAC extraction from total skin samples

Each skin sample (n = 3; 2 cm²) was spiked with a certain concentration of TAC (in acetone). After acetone evaporation, skin samples were cut into small pieces and soaked in 4 mL of ACN with internal standard (PIM) and let overnight. The skin extracts were then analyzed by UHPLC-MS/MS and calibration curve was prepared in ACN. The procedure extraction of total skin was validated as presented in Table S9.

Table S9. Validation of TAC extraction from total skin samples.

**Applied amount/cm² (μg)**	**Recovered amount/cm² (Mean ± SD in μg)**	**Recovery (%)**
20.0	16.57 ± 1.23	86.15
10.0	9.29 ± 0.29	92.21
0.05	0.06 ± 0.01	110.16

For all skin samples more than 85% of TAC was recovered during the extraction procedure.

Validation of TAC extraction form sliced skin samples

Each skin sample was snap-frozen in isopentane cooled to its freezing point (-160°C) with liquid nitrogen and mounted in the cryotome. The samples were sliced so as to obtain 40 μm slices. Each slice (n = 3; 0.8 cm²) was spiked with a certain concentration of TAC (in acetone). After acetone evaporation, slices were extracted in 300 μL of ACN with internal standard (PIM) and let overnight. The skin extracts were

then analyzed by UHPLC-MS/MS and a calibration curve was prepared in ACN. The procedure extraction of total skin was validated as presented in Table S10.

Table S10. Validation of TAC extraction from sliced skin samples.

Applied amount/cm^2 (ng)	Recovered amount/cm^2 (Mean ± SD in ng)	Recovery (%)
62.5	41.96 ± 5.06	88.11
9.4	8.77 ± 0.49	93.53
4.7	4.09 ± 0.31	87.32

For all skin samples more than 85% of TAC was recovered during the extraction procedure.

Analytical method for SIR

Validation of UHPLC-UV method

Specificity

The method was considered specific for SIR. Indeed, SIR was eluted at 0.48 min and the peaks due to the polymer appeared at 1.41 min (for TPGS) and at 2.23 min (for α-tocopherol). The chromatograms in Figure S9 present the signals of SIR standard (30 µg/mL), ACN blank, SIR loaded in micelles and unloaded micelles.

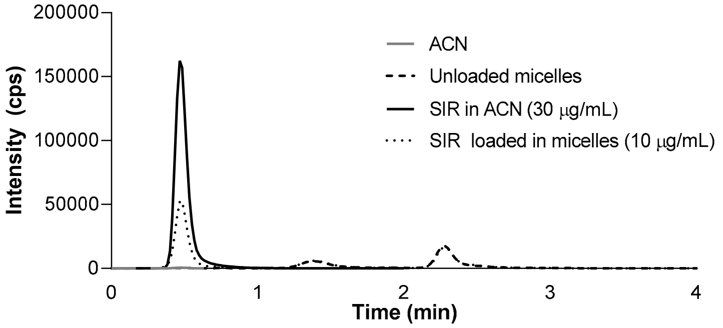

Figure S9. Chromatograms of ACN blank, unloaded micelles, SIR standard (30 µg/mL) and SIR loaded in micelles.

Linearity

Calibration curves were constructed by plotting SIR peak area (cps/min) against SIR nominal concentration (ng/mL). A good linear fit was found in the concentration range of 1 – 100 µg/mL. Correlation coefficients for all calibration curves were superior to 0.99.

Limit of detection and limit of quantification

The limit of detection (LOD) and the limit of quantification (LOQ) of SIR were found to be 1 and 3µg/mL, respectively.

Precision and accuracy

Intra- and inter-day precision and accuracy were tested using 3, 10, 50 µg/mL of SIR in ACN. Table S11 presents the results obtained for intra- and inter-day precision and accuracy.

Table S11. Intra- and inter-day precision and accuracy for quantification of SIR in ACN with UHPLC-UV.

[SIR]$_{theo}$ (μg/mL)	Intra-day [SIR]$_{meas}$ (μg/mL)	RSD (%)	Recovery (%)	Inter-day 1 [SIR]$_{meas}$ (μg/mL)	RSD (%)	Recovery (%)	Inter-day 2 [SIR]$_{meas}$ (μg/mL)	RSD (%)	Recovery (%)
3	2.80 ± 0.05	1.92	93.4	3.10 ± 0.04	1.40	103.5	2.98 ± 0.02	0.65	99.5
10	9.79 ± 0.06	0.56	97.9	10.05 ± 0.06	0.64	100.5	10.16 ± 0.14	1.42	101.6
50	49.54 ± 0.22	0.45	99.1	49.95 ± 0.19	0.38	99.9	51.07 ± 0.53	1.04	102.1

The results indicated that for intra-day measurements, the mean recoveries ranged from 93.4 to 99.1 (RSD 0.45 – 1.92%). The mean recoveries for inter-day analysis on day 1 were between 99.9 and 103.5 (RSD 0.38 – 1.40%) and on day 2, 99.5 and 102.1 (RSD 0.65 – 1.42%). Considering the ICH (2005) and FDA Bioanalytical Method Validation (2001) guidelines, the method was considered accurate and precise.

Validation of UHPLC-MS/MS method in ACN

Specificity

The chromatograms in Figure S10 present the MS signal of SIR and SIR-D3 standard alone and mixture of the compounds prepared in ACN.

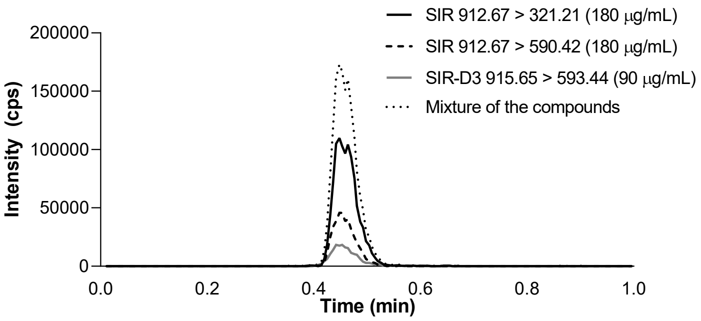

Figure S10. Respective MRM traces on XEVO TQ MS of **a)** SIR 912.67 > 321.21, **b)** SIR 912.67 > 590.42, **c)** SIR-D3 915.65 > 593.44 and **d)** mixture of compounds prepared in ACN.

Linearity

Calibration curves were constructed by plotting the ratio of SIR and SIR-D3 peak area (cps/min) against the ratio of SIR and SIR-D3 nominal concentration (ng/mL). A good linear fit was found in the concentration range of 0.01 – 2.5 (SIR/SIR-D3 concentration ratio), that corresponds from 225 to 1 ng/mL of SIR. Correlation coefficients for all calibration curves were superior to 0.99.

Limit of detection and limit of quantification

The limit of detection (LOD) and the limit of quantification (LOQ) of SIR were found to be 0.3 and 1 ng/mL, respectively.

Precision and accuracy

Intra- and inter-day precision and accuracy were tested using 1, 12, 120 ng/mL of SIR in ACN. Table S12 presents the results obtained for intra- and inter-day precision and accuracy.

Table S12. Intra- and inter-day precision and accuracy for quantification of SIR in ACN with XEVO TQ MS.

	Intra-day			Inter-day 1			Inter-day 2		

[SIR]theo (ng/mL)	[SIR]meas (ng/mL)	RSD (%)	Recovery (%)	[SIR]meas (ng/mL)	RSD (%)	Recovery (%)	[SIR]meas (ng/mL)	RSD (%)	Recovery (%)
1	0.87 ± 0.05	5.75	87.9	0.93 ± 0.13	13.57	96.1	1.01 ± 0.12	12.21	100.9
12	11.05 ± 0.22	1.97	92.0	12.24 ± 0.65	5.34	102.0	12.54 ± 0.69	5.54	104.5
120	118.29 ± 4.10	3.46	98.6	114.28 ± 2.03	1.77	95.2	116.27 ± 1.84	1.59	96.9

The results indicated that for intra-day measurements, the mean recoveries ranged from 87.9 to 98.6 (RSD 1.97 – 5.75%). The mean recoveries for inter-day analysis on day 1 were between 95.2 and 102.0 (RSD 1.77 – 13.57%) and on day 2, 96.9 and 104.5 (RSD 1.59 – 12.21%). Considering the ICH (2005) and FDA Bioanalytical Method Validation (2001) guidelines, the method was considered accurate and precise.

Validation of UHPLC-MS/MS method in skin matrix

Specificity

The chromatograms in Figure S11 present the MS signal of SIR, SIR-D3 and mixture of the compounds prepared in skin matrix.

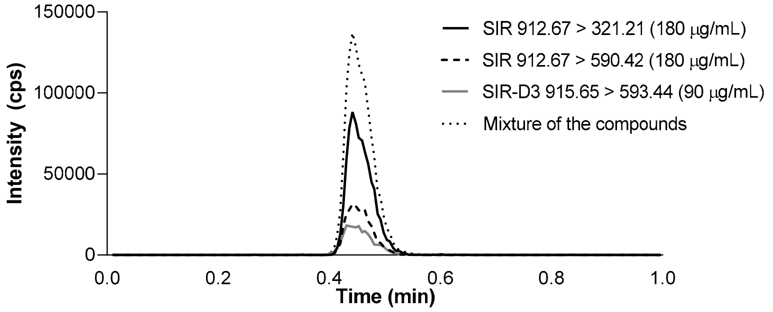

Figure S11. Respective MRM traces on XEVO TQ MS of **a)** SIR 912.67 > 321.21, **b)** SIR 912.67 > 590.42, **c)** SIR-D3 915.65 > 593.44 and **d)** mixture of compounds prepared in skin matrix.

Figure S12 presents the chromatograms obtained for SIR standard, blank permeation and blank extraction sample. No SIR was found in blank skin permeation and extraction samples.

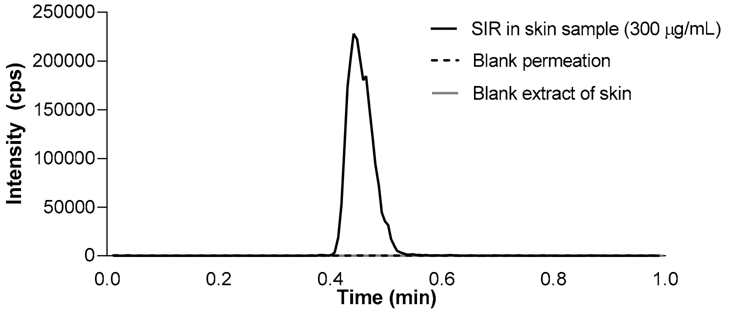

Figure S12. Respective MRM traces of SIR in skin samples (912.67 > 590.42 and 321.29): **a)** SIR in matrix, **b)** blank permeation and **c)** blank extract of skin.

The method was considered as specific for SIR quantification in skin samples.

Linearity

Calibration curves were constructed by plotting the ratio of SIR and SIR-D3 peak area (cps/min) against the ratio of SIR and SIR-D3 nominal concentration (ng/mL). A good linear fit was found in the concentration range of 0.03 – 2.5 (SIR/SIR-D3 concentration ratio), that corresponds from 225 to 3 ng/mL of SIR. Correlation coefficients for all calibration curves were superior to 0.99.

Limit of detection and limit of quantification

The limit of detection (LOD) and the limit of quantification (LOQ) of SIR were found to be 1 and 3 ng/mL, respectively.

Precision and accuracy

Intra- and inter-day precision and accuracy were tested using 3, 12, 120 ng/mL of SIR in ACN. Table S13 presents the results obtained for intra- and inter-day precision and accuracy.

Table S13. Intra- and inter-day precision and accuracy for quantification of SIR in skin matrix with XEVO TQ MS.

	Intra-day			Inter-day 1			Inter-day 2		
$[SIR]_{theo}$ (ng/mL)	$[SIR]_{meas}$ (ng/mL)	RSD (%)	Recovery (%)	$[SIR]_{meas}$ (ng/mL)	RSD (%)	Recovery (%)	$[SIR]_{meas}$ (ng/mL)	RSD (%)	Recovery (%)
3	3.26 ± 0.16	4.90	108.7	2.61 ± 0.18	6.88	86.9	3.18 ± 0.41	12.99	107.8
12	12.32 ± 0.64	5.24	102.7	11.73 ± 0.89	7.60	97.7	11.84 ± 0.74	6.26	98.6
120	112.41 ± 3.35	2.98	93.7	112.90 ± 3.02	2.68	94.1	112.47 ± 5.19	4.61	93.7

The results indicated that for intra-day measurements, the mean recoveries ranged from 93.7 to 108.7 (RSD 2.98 – 5.24%). The mean recoveries for inter-day analysis on day 1 were between 86.9 and 97.7 (RSD 2.68 – 7.60%) and on day 2, 93.7 and 107.8 (RSD 4.61 – 12.99%). Considering the ICH (2005) and FDA Bioanalytical Method Validation (2001) guidelines, the method was considered accurate and precise.

Validation of skin extraction procedure

The ability of the extraction methods to recover the entire SIR deposited amount during *in vitro* permeation experiments was tested.

Validation of SIR extraction from total skin samples

Each skin sample (n = 3; 2 cm^2) was spiked with a certain concentration of SIR (in acetone). After acetone evaporation, skin samples were cut into small pieces and soaked in 4 mL of ACN with internal standard (SIR-D3) and left overnight. The skin extracts were then analyzed by UHPLC-MS/MS and calibration curve was prepared in ACN. The procedure extraction of total skin was validated as presented in Table S14.

Table S14. Validation of SIR extraction from total skin samples.

Applied amount/cm^2 (μg)	Recovered amount/cm^2 (Mean ± SD in μg)	Recovery (%)
14	14.86 ± 0.86	106.10
4	4.21 ± 0.29	105.33
0.14	0.13 ± 0.01	94.95

For all skin samples more than 95% of SIR was recovered during the extraction procedure.

Validation of SIR extraction from sliced skin samples

Each skin sample was snap-frozen in isopentane cooled to its freezing point (-160°C) with liquid nitrogen and mounted in the cryotome. The samples were sliced so as to obtain 40 µm slices. Each slice (n = 3; 0.8 cm^2) was spiked with a certain concentration of SIR (in acetone). After acetone evaporation, slices were extracted in 300 µL of ACN with internal standard (SIR-D3) and left overnight. The skin extracts were then analyzed by UHPLC-MS/MS and calibration curve was prepared in ACN. The procedure extraction of total skin was validated as presented in Table S15.

Table S15. Validation of SIR extraction from sliced skin samples.

Applied amount/cm^2 (ng)	Recovered amount/cm^2 (Mean ± SD in ng)	Recovery (%)
50	51.27 ± 1.94	84.05
5	6.04 ± 0.40	96.67
2	2.49 ± 0.38	99.58

For all skin samples more than 90% of SIR was recovered during the extraction procedure.

Micelle size

Table S16 summarizes the micelle size obtained for the six formulations loaded with the immunosuppressive drugs.

Table S16. Micelle size expressed in terms of number-weighted diameters for the six formulations.

	**d_N (nm) [%] d_N**		
	Sirolimus	Pimecrolimus	Tacrolimus
Formulation 1	8.02 [100%]	8.56 [100%]	7.42 [100%]
Formulation 2	7.94 [100%]	8.23 [100%]	8.12 [100%]
Formulation 3	8.20 [100%]	8.13 [100%]	7.86 [100%]
Formulation 4	8.09 [100%]	7.67 [100%]	7.41 [100%]
Formulation 5	7.43 [100%]	7.94 [100%]	7.28 [100%]
Formulation 6	7.63 [100%]	7.55 [100%]	7.43 [100%]
